# Huwe1 interacts with Gadd45b under oxygen-glucose deprivation and reperfusion injury in primary Rat cortical neuronal cells

**DOI:** 10.1186/s13041-015-0178-y

**Published:** 2015-12-23

**Authors:** Guo-qian He, Wen-ming Xu, Jin-fang Li, Shuai-shuai Li, Bin Liu, Xiao-dan Tan, Chang-qing Li

**Affiliations:** Department of Neurology, The Second Affiliated Hospital of Chongqing Medical University, Chongqing, 400010 China; Department of Obstetrics and Gynecology, Joint Laboratory of Reproductive Medicine, Sichuan University-The Chinese University of Hongkong Joint Laboratory for Reproductive Medicine (SCU-CUHK), Institute of Women and Children’s Health, West China Second University Hospital, Sichuan University, Chengdu, 610041 China; Department of Neurology, Shandong Provincial Qianfoshan Hospital, Jinan, 250000 China

**Keywords:** Gadd45b, Huwe1, BDNF, Oxygen-glucose deprivation and reperfusion, Methylation

## Abstract

**Background:**

Growth arrest and DNA-damage inducible protein 45 beta (Gadd45b) is serving as a neuronal activity sensor. Brain ischemia induces the expression of Gadd45b, which stimulates recovery after stroke and may play a protective role in cerebral ischemia. However, little is known of the molecular mechanisms of how Gadd45b expression regulated and the down-stream targets in brain ischemia. Here, using an oxygen-glucose deprivation and reperfusion (OGD/R) model, we identified Huwe1/Mule/ARF-BP1, a HECT domain containing ubiquitin ligase, involved in the control of Gadd45b protein level. In this study, we also investigated the role of Huwe1-Gadd45b mediated pathway in BDNF methylation.

**Results:**

We found that the depletion of Huwe1 by lentivirus shRNA mediated interference significantly increased the expression of Gadd45b and BDNF at 24 h after OGD. Moreover, treatment with Cycloheximide (CHX) inhibited endogenous expression of Gadd45b, and promoted expression of Gadd45b after co-treated with lentivirus shRNA-Huwe1. Inhibition of Gadd45b by lentivirus shRNA decreased the expression levels of brain derived neurotrophic factor (BDNF) and phosphorylated cAMP response element-binding protein (p-CREB) pathway, while inhibition of Huwe1 increased the expression levels of BDNF and p-CREB. Moreover, shRNA-Huwe1 treatment decreased the methylation level of the fifth CpG islands (123 bp apart from BDNF IXa), while shRNA-Gadd45b treatment increased the methylation level of the forth CpG islands (105 bp apart from BDNF IXa).

**Conclusions:**

These findings suggested that Huwe1 involved in the regulation of Gadd45b expression under OGD/R, providing a novel route for neurons following cerebral ischemia-reperfusion injury. It also indicated that the methylation of BDNF IXa was affected by Gadd45b as well as Huwe1 in the OGD/R model.

**Electronic supplementary material:**

The online version of this article (doi:10.1186/s13041-015-0178-y) contains supplementary material, which is available to authorized users.

## Backgrounds

Stroke remains a leading cause of death and disability in the world. The neurological functional disruption caused by stroke is often severe. Nevertheless, surviving patients exhibit a certain degree of restoration of function attributable to the remarkable capacity of the adult brain plasticity [1, 2]. In order to potentiate post-stroke recovery, several rehabilitation therapies have been undertaken [3, 4].

Growth arrest and DNA-damage inducible protein 45 beta (Gadd45b), originally known as MyD118, is a member of the highly homologous Gadd45 family of proteins including Gadd45a, b, and g [5]. More recently, Gadd45b was shown as a neuronal activity sensor, essential for adult neurogenesis by demethylating and activating key neurogenic genes like brain-derived neurotrophic factor (BDNF) [6]. This discovery prompted a growing body of literature documenting their role as players in adult cognitive function and central nervous system diseases, such as electroconvulsive seizure [6, 7], and psychotic disorders [8]. Recently, our study has reported that rat brain ischemia stimulated the expression of Gadd45b in the cortex, Gadd45b-RNAi significantly decreased the level of BDNF and inhibited axonal plasticity after MCAO [9]. Our results showed that Gadd45b stimulated recovery after stroke, and may play a protective role in cerebral ischemia [9, 10]. However, little is known about the molecular mechanisms for how the expression of Gadd45b is regulated in brain ischemia. Furthermore, the mechanism for the Gadd45b effect on BDNF under brain ischemia is still poorly understood.

The ubiquitin-proteasome system (UPS) as the major intracellular machinery for protein degradation, has been attracted more attention in neurobiology [11]. The accumulation of ubiquitin-containing protein aggregates following cerebral ischemia is a general feature [12]. Previous studies have showed that the major control and selectivity are determined by ubiquitin E3 ligase at the substrate ubiquitination step [13]. The HECT-domain E3 ligase Huwe1 (HECT, UBA and WWE domain containing 1) is involved in the ubiquitination, degradation of multiple proteins, playing diverse biological roles and the altered expression is detected in multiple diseases [14–17]. Huwe1 is important for neurogenesis in cerebral cortex and possibly brain cancer, thus plays critical roles in nervous system plasticity, regeneration and disease [14]. Previous studies have indicated that Gadd45 is ubiquitinated and regulated by proteolysis [18, 19]. Proteasome inhibitor MG132 increased the expression of Gadd45b in prostate cancer cells [19].

Based on these studies, given that the expression of Gadd45b is affected by UPS, we hypothesize that Huwe1 might play an important role in regulating Gadd45b expression in brain ischemia. And Gadd45b maybe affect the level of BDNF by demethylation of CpG islands in brain ischemia. To verify the hypothesis, we used the oxygen glucose deprivation and reperfusion (OGD/R) model of rat cortical neuronal cells to mimic cerebral ischemia-reperfusion condition in vitro. Therefore, we examined the effects of Huwe1 on Gadd45b and BDNF signaling during ischemia-reperfusion injury. We also examined the effects of Gadd45b on BDNF signaling, and characterized the effects of Huwe1 and Gadd45b on BDNF methylation as well as down-stream targets.

## Results

### Expression of Huwe1 and Gadd45b following OGD/R

**Fig. 1:**
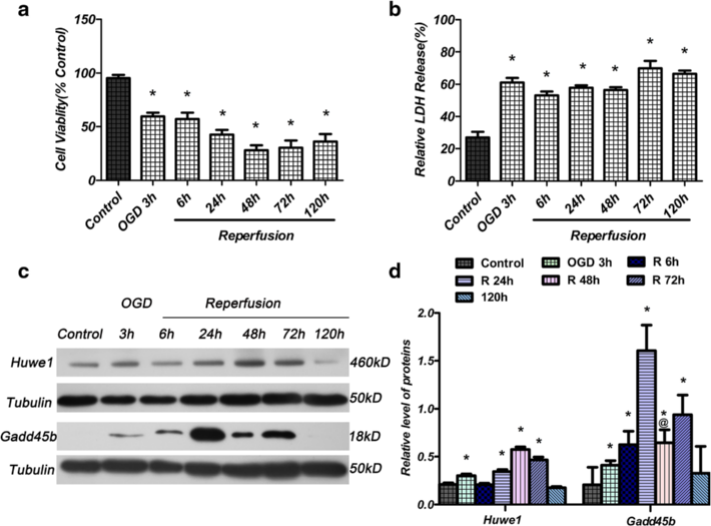
Expression of Huwe1 and Gadd45b in cortical neuronal cells after OGD/R. Primary cortical neurons were subjected to 3 h OGD followed by reperfusion for 6 h, 24 h, 48 h, 72 h and 120 h. **a** Oxygen glucose deprivation and reperfusion decreased the cell viability, compared to normal neuron cells control. Cell viability was analyzed by CCK-8 assay. **b** Oxygen glucose deprivation and reperfusion induced cortex neuron cells death. Cell death was evaluated by LDH assay. **c** Western blot assay of Huwe1 and Gadd45b expression at the indicated time points after OGD/R. **d** The quantitative results from (*c*). Tubulin was used as an internal control. *p < 0.05 versus *control.* ^*@*^ *p < 0.05* versus *OGD/R 24 h*

We subjected primary cortical neurons to 3 h of OGD followed by reperfusion for 0, 6, 24, 48, 72 and 120 h, and then measured OGD/R-induced neuronal cell viability reduction and LDH release. As shown in Fig. 1a, neuronal cell viability declined progressively as the reperfusion time prolonged. About only 42.5 % of neuron cells remained viable at 24 h. Meanwhile, LDH leakage was markedly increased from OGD 3 to 72 h after reperfusion (Fig. 1b).

To examine the effect of Huwe1 on Gadd45b under OGD/R, we then analyzed the expression of Huwe1 and Gadd45b by western blot in both normal neurons and OGD/R-treated neurons at different time points. As shown in Fig. 1c, western blot analysis showed that the expression of Huwe1 increased from OGD 3 to 72 h after reperfusion compared to controls, and reached a peak at 48 h after reperfusion. However, the expression of Gadd45b increased since OGD 3 h and lasted up to 72 h, and reached a peak at 24 h. There was a down trending of the expression of Gadd45b at 48 h. We assumed that there may be a correlation between Huwe1 and Gadd45b at 24 h after OGD, so we choose this time point for further study.

### Inhibition of Huwe1 increased the expression of Gadd45b

**Fig. 2:**
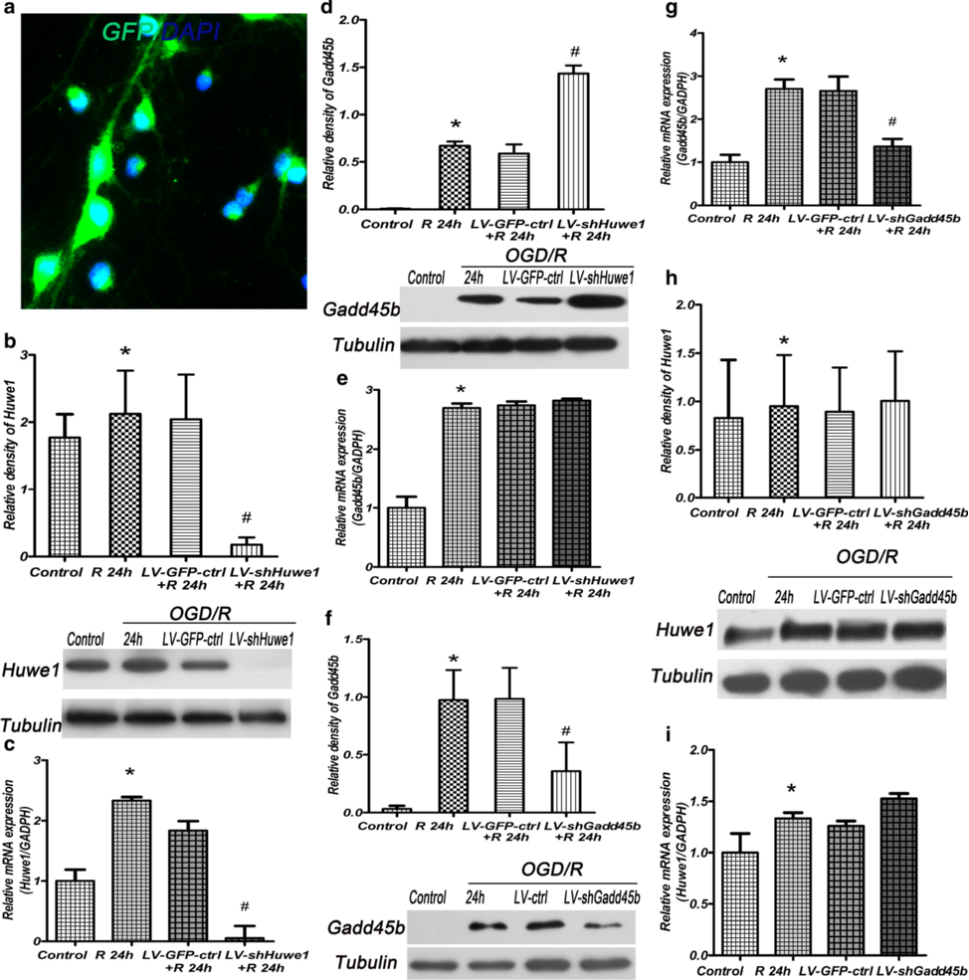
Inhibition of Huwe1 increased the expression of Gadd45b at 24 h after reperfusion. Neuron cells were treated with lentivirus shRNA-Huwe1 or shRNA-Gadd45b on DIV 3, and then exposed to OGD 3 h and reperfusion for 24 h on DIV 7. Cells were prepared for western blot and qPCR. **a** The GFP-expressing lentiviral vectors cells were observed under an invert fluorescent microscope. It showed GFP (green) expressed in neurons. DAPI (blue) was used for cell nucleus staining. **b** ShRNA-Huwe1 (LV-shHuwe1) obviously decreased the protein expression of Huwe1 at 24 h, compared to the lentivirus-GFP-scramble control group (LV-GFP-ctrl), as seen by western blotting. **c** ShRNA-Huwe1 also decreased the mRNA level of Huwe1 at 24 h, as seen by qPCR. **d** ShRNA-Huwe1 increased the protein expression of Gadd45b at 24 h. **e** ShRNA-Huwe1 had no effect on the mRNA level of Gadd45b at 24 h. **f** ShRNA-Gadd45b (LV-shGadd45b) decreased the protein expression of Gadd45b at 24 h compared to the lentivirus-scramble control group (LV-ctrl). **g** ShRNA-Gadd45b decreased the mRNA level of Gadd45b at 24 h. **h**-**i** ShRNA-Gadd45b did not affect the protein and mRNA levels of Huwe1 at 24 h, as seen by western blotting (**h**) and qPCR (**i**). Tubulin was used as an internal control for western blot; GADPH was used as an internal control for qPCR. *p < 0.05 versus *control,* ^***#***^ *p < 0.05* versus *LV-GFP-ctrl or LV-ctrl. Scale bar = 10 μm*

To further determine whether Huwe1 was involved in the regulation of Gadd45b after OGD/R, shRNA-Huwe1 or shRNA-Gadd45b was added to the culture medium on DIV 3. As shown in Fig. 2b-c, treatment with shRNA-Huwe1 significantly decreased the protein and mRNA levels of Huwe1 at 24 h after OGD, compared to lentivirus-GFP-scramble control group. Conversely, shRNA-Huwe1 increased the protein level of Gadd45b at 24 h, compared to lentivirus-GFP-scramble control group (Fig. 2d). The mRNA level of Gadd45b was not changed at 24 h after treatment with shRNA-Huwe1 (Fig. 2e). Treatment with shRNA-Gadd45b reduced the protein and mRNA levels of Gadd45b at 24 h, compared to lentivirus-scramble control group (Fig. 2f, g). However, shRNA-Gadd45b did not affect the expression of Huwe1 at 24 h, compared to lentivirus-scramble control group (Fig. 2h, i). Moreover, we used another probe sequence for shRNA targeted to the coding sequence of Huwe1, and found that shRNA-Huwe1-2 also increased the expression of Gadd45b at 24 h after OGD/R (Additional file 1: Figure S1). The effect of the shRNA-Huwe1-1 or shRNA-Huwe1-2 on basal level of Huwe1 and Gadd45b was also examined in the normal neuron cells without OGD/R (Additional file 1: Figure S1).

### Huwe1 interacts with Gadd45b

**Fig. 3:**
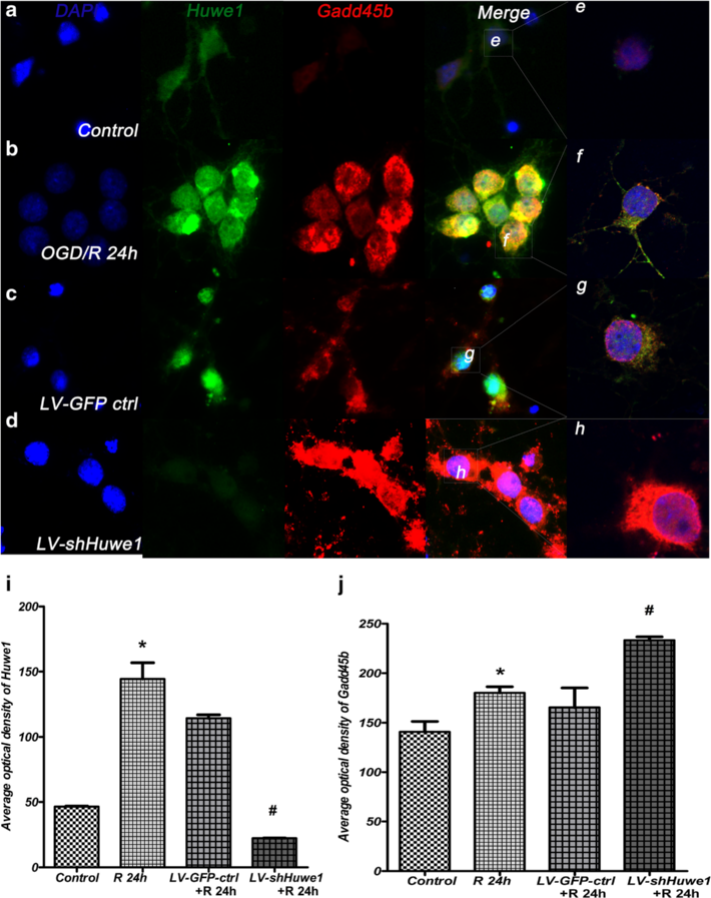
The co-expression of Huwe1 and Gadd45b existed in cortex neuron cells. Cells were prepared to incubate with anti-Huwe1 (Green) and anti-Gadd45b (Red) antibodies for double immunofluorescent labeling staining. DAPI (blue) was used for cell nucleus staining. The co-expression of Huwe1 and Gadd45b obviously increased at 24 h after reperfusion (**b**), compared to normal neuron cells controls (**a**). Inhibition of Huwe1 (**d**) increased the expression of Gadd45b, compared to the lentivirus-GFP-scramble control group (**c**) at 24 h. (**e**-**h**) showed that the co-localization of Huwe1 and Gadd45b by laser confocal scanning microscopy. The co-localization of Huwe1 and Gadd45b is mainly around the cell nucleus (**f**-**g**). **i**-**j** The average optical density results of Huwe1 and Gadd45b. *p < 0.05 versus *control,* ^*#*^ *p < 0.05* versus *LV-GFP-ctrl. Scale bar = 10 μm*

Based on these data, we examined the co-expression of Huwe1 and Gadd45b in cortex neuron cells by fluorescent dual-labeling method. As shown in Fig. 3a, there was little co-expression of Huwe1 and Gadd45b in normal cortex neuron cells. However, at 24 h after reperfusion, the co-expression of Huwe1 and Gadd45b was obviously increased (Fig. 3b). The co-expression of Huwe1 and Gadd45b was mainly around the nucleus (Fig. 3f) by Confocal Fluorescence Microscopy. Treatment with shRNA-Huwe1 increased the optical density of Gadd45b (Fig. 3d), compared to treatment with lentivirus-GFP-scramble control group (Fig. 3c). The immunofluorescent staining results of treatment with shRNA-Huwe1 were similar to the results of western blot (Fig. 3i, j).

**Fig. 4:**
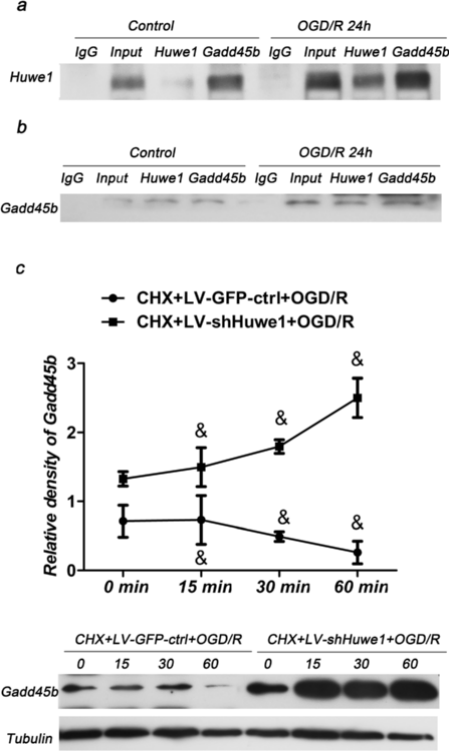
Huwe1 interacted with Gadd45b at 24 h after reperfusion. **a**, **b** Protein was extracted from normal neuron cells or neuron cells exposed to OGD/R 24 h, and prepared from CO-IP assay. Protein was incubated with IgG antibody, as a negative control (IgG). Protein was not incubated with antibodies, as a positive control (Input); Protein was also incubated with anti-Huwe1 antibody (**a**) or anti-Gadd45b antibody (**b**), followed by western blot. Note that the interaction of Huwe1 and Gadd45b at 24 h after reperfusion compared to normal neuron cells. **c** Co-treatment with CHX and lentivirus-GFP-scramble stably decreased the expression of Gadd45b since 0–60 min at 24 h after reperfusion. Conversely, co-treatment with CHX and shRNA-Huwe1 stably increased the expression of Gadd45b since 0–60 min. *Tubulin was used as an internal control for western blot; &p < 0.05* versus *LV-GFP-ctrl at different time point for CHX*

To evaluate whether Huwe1 interacted with Gadd45b, neurons were subjected to co-immunoprecipitation assay. The reciprocal co-immunoprecipitation verified this interaction at 24 h after reperfusion, compared to normal neuron cells (Fig. 4a). We then determined the effects of Huwe1 on the protein stability of Gadd45b at defined intervals after treatment with CHX to block de novo protein synthesis. CHX obviously inhibited the expression of Gadd45b at 24 h (Fig. 4c). Conversely, co-treatment with shRNA-Huwe1 with CHX stably increased the expression of Gadd45b at 24 h (Fig. 4c). These results demonstrated that Huwe1 may promote the protein degradation of Gadd45b.

### Inhibition of Gadd45b significantly reduced the expression of BDNF

**Fig. 5:**
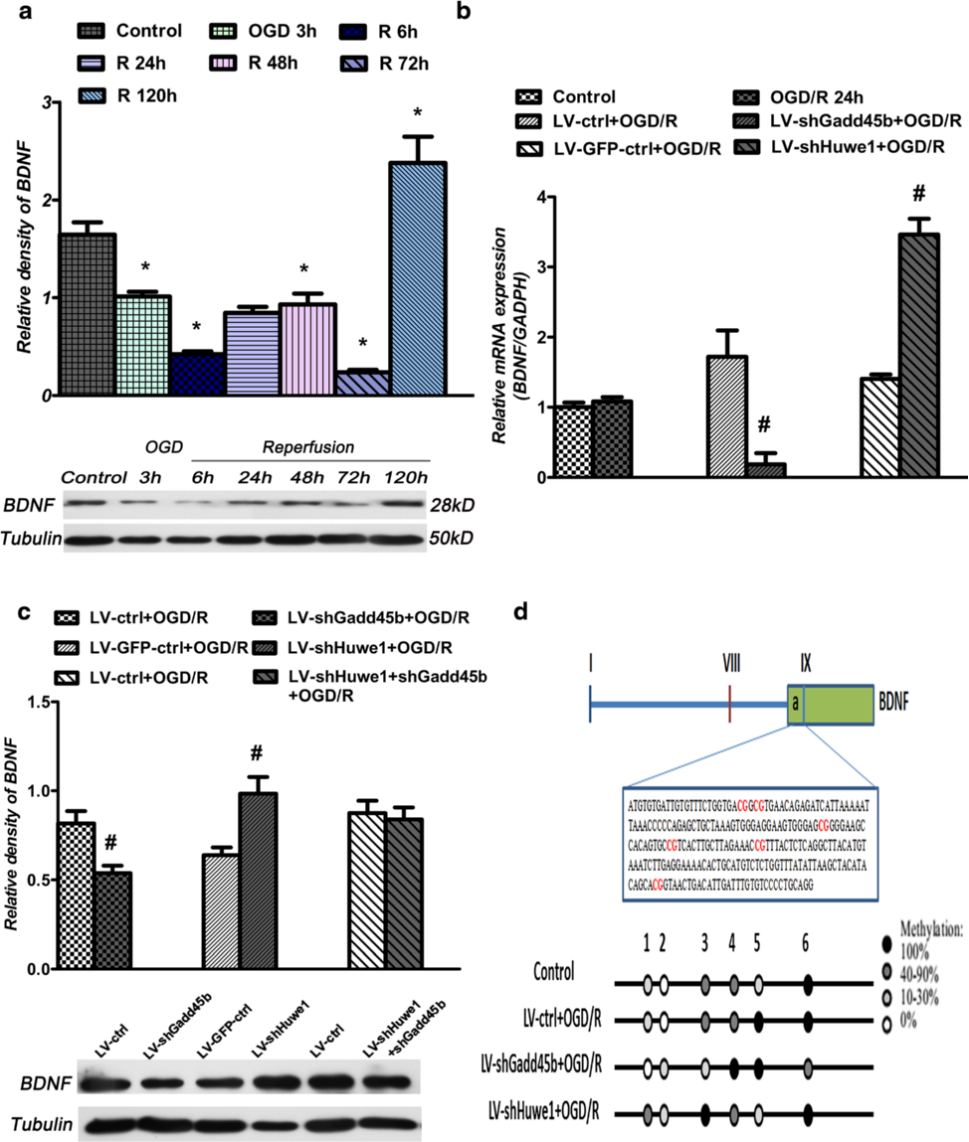
Inhibition of Gadd45b decreased the expression of BDNF at 24 h after reperfusion. **a** Western blot assay of BDNF expression at the indicated time points after OGD/R. **b** Neuron cells were infected with lentivirus shRNA-Huwe1 and/or lentivirus shRNA-Gadd45b, followed by OGD and reperfusion 24 h. ShRNA-Gadd45b (LV-shGadd45b) decreased the expression of BDNF, compared to lentivirus scramble control group (LV-ctrl). However, shRNA-Huwe1 (LV-shHuwe1) increased the expression of BDNF. **c** *qPCR result of BDNF after treatment with shRNA-Huwe1 or shRNA-Gadd45b.* **d** The methylation results of BDNF IXa by BSP assay. The BDNF IXa included six CpG islands. Inhibition of Huwe1 or Gadd45b changed the level of BDNF IXa methylation at 24 h after reperfusion. Tubulin was used as an internal control for western blot; GADPH was used as an internal control for qPCR; *p < 0.05 versus *control,* ^***#***^ *p < 0.05* versus *LV-GFP-ctrl or LV-ctrl*

As shown in Fig. 5a, the expression of BDNF decreased after OGD, compared to normal neuron cells control. There was a slowly increased trend from 24 h and significantly increased at 120 h after reperfusion. Fig 5b showed that shRNA-Gadd45b significantly reduced the expression of BDNF at 24 h, compared to lentivirus-scramble control group. ShRNA-Huwe1 increased the expression of BDNF at 24 h, compared to lentivirus-GFP-scramble control group. However, shRNA-Gadd45b co-treatment with shRNA-Huwe1 had no effect on the expression of BDNF. We next examined the effect of shRNA-Gadd45b and shRNA-Huwe1 on the mRNA level of BDNF (Fig. 5c). ShRNA-Gadd45b significantly reduced the mRNA level of BDNF at 24 h. Conversely, shRNA-Huwe1 increased the mRNA level of BDNF at 24 h.

Others had demonstrated that Gadd45b affects the methylation of BDNF. DNA methylation at cytosine residues occurs at CpG dinucleotides, and demethylation typically activates gene expression, which is found in high-density areas or so-called islands located within proximal promoters. Although the expression of BDNF can be initiated by nine alternative promoters (for exons I to IX), other authors had shown that only the promoter of exon IX undergoes rapid DNA demethylation upon neuronal activity induction. Previous research had demonstrated that less Gadd45b binding to the BDNF IXabcd promoter, especially the IXa, reduced the mRNA level of BDNF in psychotic subjects. So, we next examined the DNA methylation level of BDNF IXa under OGD/R. To validate the methylation status of the BDNF IXa, we designed specific PCR primers for bisulfite sequencing PCR (BSP). There are six CpG islands (24, 27, 88, 105, 123, 200 bp apart from the IXa starting nucleic acid) around BDNF IXa. As shown in Fig. 5d, the methylation level of the fifth CpG islands (123 bp) was hyper-methylated (100 %) at 24 h after reperfusion, compared to normal cortex neuron cells control. ShRNA-Huwe1 decreased obviously the methylation level of the fifth CpG (20 %) at 24 h. However, shRNA-Gadd45b increased the methylation level of the forth CpG (105 bp, 100 %) at 24 h.

**Fig. 6:**
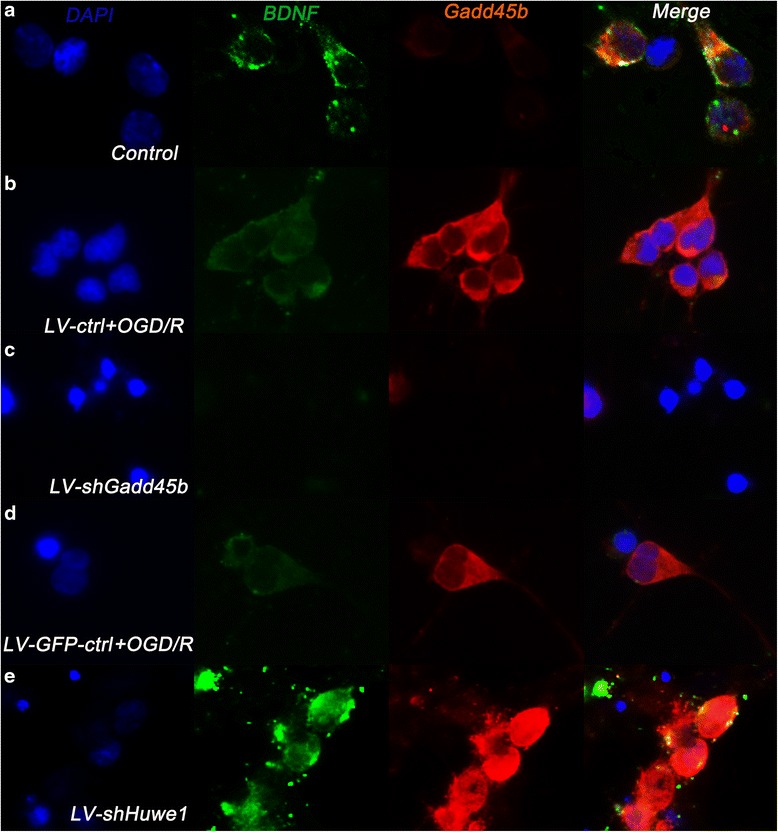
The immunofluorescent results of Gadd45b and BDNF. Neuron cells on coverslips were infected with lentivirus shRNA-Huwe1 or shRNA-Gadd45b, followed by OGD and reperfusion for 24 h. Neuron cells were incubated with the anti-Gadd45b antibody (Red) and anti-BDNF antibody (Green) for double-labeled immunofluorescence assay. The immunofluorescence of Gadd45b and BDNF is mainly around the cell nucleus in normal cortex neuron cells (**a**). Treatment with shRNA-Gadd45b (**c**) decreased the expression of Gadd45b and BDNF, compared to the lentivirus-scramble control group (**b**) at 24 h. Treatment with shRNA-Huwe1 (**e**) increased the expression of Gadd45b and BDNF, compared to the lentivirus-GFP-scramble control group (**d**) at 24 h. DAPI (blue) is used for cell nucleus staining. *Scale bar = 10 μm*

We also examined the expression of Gadd45b and BDNF in cortex neuron cells by fluorescent dual-labeling method. Figure 6 showed the double-fluorescence results of Gadd45b and BDNF, similar to the results of western blot. Treatment with shRNA-Gadd45b decreased the average optical value of Gadd45b (11.37 ± 1.74) and BDNF (3.81 ± 0.48), compared to treatment with lentivirus-scramble control group (Gadd45b: 64.92 ± 17.02, BDNF: 39.29 ± 11.27). Treatment with shRNA-Huwe1 increased the average optical value of Gadd45b (136.01 ± 26.52) and BDNF (179.01 ± 34.91), compared to treatment with lentivirus-GFP-scramble control group.

### The expression change in Gadd45b-dependent regulation of BDNF receptors and downstream

**Fig. 7:**
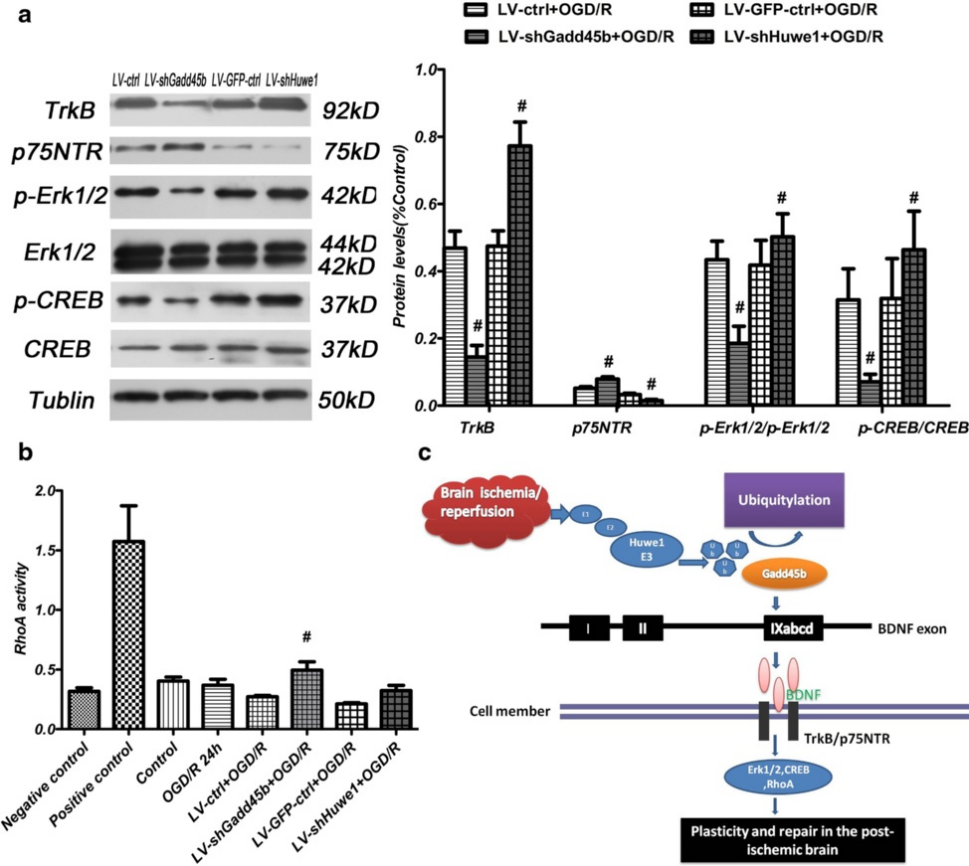
The expression of BDNF receptors and downstream at 24 h after reperfusion. Neuron cells were infected with lentivirus shRNA-Huwe1 or lentivirus shRNA-Gadd45b, followed by OGD and reperfusion 24 h. Cells were collected for western blot. **a** Inhibition of Gadd45b decreased the protein levels of TrkB, p-Erk1/2 and p-CREB and increased the expression of p75NTR, compared to the lentivirus-scramble control group (LV-ctrl). ShRNA-Huwe1 increased the expression of TrkB, p-Erk1/2 and p-CREB, and decreased the expression of p75NTR, compared to lentivirus-GFP-scramble control group (LV-GFP-ctrl). **b** Neuron cells were prepared for checking the activity of RhoA at different groups, by measuring absorbance at 490 nm using a microplate spectrophotometer. Inhibition of Gadd45b decreased the activity of RhoA. **c** The proposed scheme model for the effect of Huwe1 on Gadd45b and BDNF signal in a ubiquitination-dependent manner under oxygen-glucose deprivation and reperfusion injury in primary rat cortical neuronal cells. *Tubulin was used as an internal control for western blot;* ^***#***^ *p < 0.05* versus *LV-GFP-ctrl or LV-ctrl*

The neuroplasticity role of BDNF is restricted to its mature form, positively impacting brain function [20]. Mature BDNF once released, activates tropomyosin-related kinase B (TrkB) receptors and nerve growth factor receptor 75 (p75NTR), modulating MAPK/ERK pathway and stimulating RhoA signaling [21, 22]. As shown in Fig. 7a, treatment with shRNA-Gadd45b decreased the expression of TrkB and increased the expression of p75NTR at 24 h after reperfusion. However, shRNA-Huwe1 increased the expression of TrkB and decreased the expression of p75NTR at 24 h. ShRNA-Gadd45b decreased the rate of p-Erk1/2/Erk1/2 and p-CREB/CREB at 24 h. ShRNA-Huwe1 increased the rate of p-Erk1/2/Erk1/2 and p-CREB/CREB at 24 h. As shown in Fig. 7b, shRNA-Gadd45b increased the activity of RhoA, compared to the lentivirus-scramble control group. There was no significant difference in the activity of RhoA between the shRNA-Huwe1 group and the lentivirus-GFP-scramble control group at 24 h. However, it exhibited an increase trend toward the activity of RhoA in the shRNA-Gadd45b group. Based on our results, we proposed the scheme model for the effect of Huwe1 on Gadd45b and BDNF signaling under oxygen-glucose deprivation and reperfusion injury of primary rat cortical neuronal cells. As shown in Fig. 7c, we deduced that Huwe1 interacts with Gadd45b through ubiquitination pathway. Huwe1 and Gadd45b could regulate the expression of BDNF by way of DNA methylation, and also affect the downstream signal of BDNF.

**Fig. 8:**
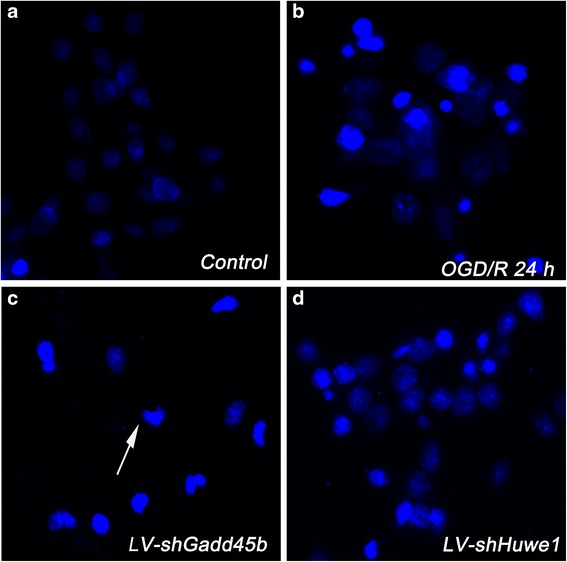
The morphological features of apoptosis were monitored by fluorescence microscopy after staining with Hoechst 33342. Neuron cells were infected with lentivirus shRNA-Huwe1 or shRNA-Gadd45b, followed by OGD and reperfusion 24 h. The dye Hoechst 33342 was introduced to characterize the cells nucleic and emits blue fluorescence at 480 nm. Cells that exhibited reduced nuclear size, chromatin condensation, intense fluorescence, and nuclear fragmentation were considered to be apoptotic (white arrow). OGD/R induced neuron cells apoptosis at 24 h (**b**), compared to normal neuron cells (**a**). Treatment with shRNA-Gadd45b increased the number of apoptotic cells (**c**). Treatment with shRNA-Huwe1 decreased the number of apoptotic cells (**d**). *Scale bar = 10 μm*

As shown in Fig. 8, the rate of cell apoptosis in the normal neuron cells was 10.97 ± 2.83. ShRNA-Gadd45b increased the rate of cell apoptosis (57.71 ± 7.49), compared to the lentivirus-control group (41.74 ± 5.54) at 24 h after reperfusion (*p* < 0.05). ShRNA-Huwe1 decreased the rate of cell apoptosis (31.40 ± 5.41), compared to the lentivirus-control group at 24 h (*p* < 0.05).

## Discussion

The major findings of this study are as follows: (1) Gadd45b and Huwe1 were activated under neuron OGD/R; (2) Inhibition of Huwe1 significantly enhanced the expression of Gadd45b under neuron OGD/R; (3) Gadd45b and Huwe1 affect the expression of BDNF through regulation of methylation under neuron OGD/R.

Transient cerebral ischemia induces irreversible protein aggregate formation. The ubiquitin-proteasome system has a central role in the selective degradation of intracellular proteins, and a particularly important role in the context of the nervous system. Previous studies showed that the UPS plays a complex and unambiguous role in the pathophysiology of cerebral ischemia-reperfusion injury [11]. While the 26S proteasome activity recovers in many regions after reperfusion (frontal cortex), in general the proteasome is irreversibly inhibited [14]. A time-dependent decrease in proteasome activity has also been detected in ipsilateral cortex during 1–24 h reperfusion after transient focal ischemia [14]. Huwe1 (also named ARF-BP1, Mule, Lasu1, Ureb1, E3 histone, and HectH9), encodes a HECT domain ubiquitin ligase, and may play critical roles in nervous system plasticity, regeneration and disease. Zhao et al. conditionally deleted the Huwe1 gene in the mouse brain and demonstrated that this enzyme is essential for the transition from self-renewing and proliferating neural stem progenitor cells to differentiated neurons [23]. In our study, we found that the expression of Gadd45b reached a peak at 24 h, and showed a decreased trend at 48 h. The expression of Huwe1 was increased from 24 to 48 h after reperfusion. Gadd45 has been demonstrated that is ubiquitinated and regulated by proteolysis previously. Although Gadd45b expression precedes Huwe1 increases, there may be a correlation between Huwe1 and Gadd45b. Huwe1 has several disparate substrates, such as p53, Mcl-1, Cdc6, and N-myc. Gadd45b may be a substrate of Huwe1. It may due to the relative low level of Huwe1 at 24 h and the relative high level at 48 h. In our study, we found that shRNA-Huwe1 effected the expression of Gadd45b at 24 h after reperfusion.

The expression of Gadd45b is low in brain at resting condition [24]. Brain ischemia induced the expression of Gadd45b. A key question is that whether the increased Gadd45b level is lasting or transient. We found the expression of Gadd45b increased after 3 h OGD, reached a peak at 24 h, and decreased to the normal level at 120 h after reperfusion. Therefore, the high expression of Gadd45b is transient in our experiment condition. Previous data suggested a model in which seizure or physiological activity stimulates neurogenesis in a paracrine manner via transient actions of Gadd45b on mature neurons [14, 24]. Short-lived Gadd45b expression leads to reversible epigenetic modifications involving demethylation of specific gene regulatory regions, both transient events resulting in more long-lasting changes in cell proliferation [24]. Multiple studies have shown that Gadd45b may be important specifically for the activity-dependent of the BDNF gene promoter demethylation, leading to subsequent increases in gene and protein expression and plasticity-related signaling [25, 26]. Less Gadd45b binding to the BDNF IXabcd promoter and reduced BDNF mRNA expression in psychotic subjects [8, 25]. Electroconvulsive seizures induced the regulatory region IX of the BDNF promoter which showed significant demethylation [24]. The time course of BDNF demethylation paralleled induction of Gadd45b after electroconvulsive seizures, both being transient and reversible. Previous study demonstrated that rapid de novo DNA methylation takes place after expression of Gadd45b decreases, within 24 h after ECT [6, 24]. In our study, the BSP results showed that shRNA-Huwe1 obviously decreased the methylation level of the fifth (123 bp apart from BDNF IXa) CpG islands (20 %) at 24 h. While, shRNA-Gadd45b increased another CpG island, which is about 105 bp apart from BDNF IXa, and hypermethylated (100 %) at 24 h after reperfusion. We found no significant change on others of the six CpG islands. Our western blot also showed that shRNA-Gadd45b decreased the expression of BDNF, but shRNA-Huwe1 increased the expression of BDNF at 24 h after reperfusion. Our study shows that in the pathological process of neuron OGD/R, methylation maybe one way of Gadd45b influencing on BDNF. Whether the effect of Gadd45b on the methylation of BDNF is long lasting or transient under OGD/R is remaining worthy of study.

BDNF binds with high specificity to the TrkB and to the low-affinity neurotrophin receptor p75 [4]. Mature BDNF activates TrkB receptors, and activated MAPK/ERK, thereby positively impacting brain function [20]. Recently, increasing evidence suggests that collapses neurites outgrowth by activating RhoA through the p75NTR signaling pathway. When neuron cells were treated with shRNA-Gadd45b, the expression of TrkB, p-Erk1/2 and p-CREB were decreased at 24 h after reperfusion. ShRNA-Huwe1 increased the expression of TrkB, p-Erk1/2 and p-CREB at 24 h. This indicated that Gadd45b and Huwe1 affected the BDNF and BDNF downstream effectors. Activation of the Erk signaling pathway has been reported to regulate neuronal differentiation [4]. Moreover, previously, we have reported that Gadd45b-RNAi significantly inhibited axonal plasticity after rat MCAO, and also significantly decreased the expression levels of both BDNF and p-CREB pathway and promoted ROCK expression at 14 days after MCAO [9]. In our study, we also found shRNA-Gadd45b affected the RhoA. This indicated that transient action of Gadd45b may lead to the lasting affect after brain ischemia. Moreover, we found that Huwe1 and Gadd45b affected apoptosis under OGD/R. On the basis of our results, we proposed the scheme model for the effect of Huwe1 on Gadd45b and BDNF signaling. Gadd45b may be a substrate of Huwe1 under OGD/R. Huwe1 and Gadd45b affected the expression of BDNF by way of BDNF IXa methylation, and then affected the expression of BDNF receptors and downstream molecules, thereby took apart in the plasticity and repair under OGD/R. However, the scheme model yet needs further studies and more verification in all aspects.

## Conclusion

This study suggested that a negative association between Gadd45b and Huwe1. Huwe1 may regulate Gadd45b protein stability in cerebral OGD/R injury. Gadd45b and Huwe1 may regulate the expression of BDNF via BDNF IXa methylation. Further studies are needed to delineate the precise role of Gadd45b in ischemic recovery and to investigate the therapeutic potential of targeting their expression and function.

## Methods

### Antibodies and reagents

Neuron cell culture medium and reagents were obtained from Life Technologies. Poly-D-lysine (PDL, #P6407), Cycloheximide (CHX, #R750107), Cytotoxicity Detection Kit (LDH, #11644793001), and Hoechst 33342 (#B2261) were obtained from Sigma. Cell Counting Kit-8 assay (CCK-8, #CK04) was obtained from Dojindo. Magnetic Beads (#2033657) and anti-IgG were purchased from Millipore. G-LISA^®^ RhoA Activation Assay Biochem Kit™ (#BK124) was purchased from Cytoskeleton. Rabbit monoclonal antibody against Huwe1 (1:700; #LS-B1359) was purchased from Lifespan biosciencesinc. Rabbit monoclonal antibodies against Gadd45b (1:10000; #ab128920), BDNF (1:1000; #ab108383), p75 (1:20000; #ab52987), CREB (1:1000; #ab32515), phospho-S133-CREB (p-CREB, 1:500; #ab32096) were purchased from Abcam; Rabbit polyclonal antibody against TrkB (1:1000; #ab18987) was purchased from Abcam; Rabbit monoclonal antibodies against phospho-Thr202/Tyr204-ERK1/2 (p-ERK1/2, 1:2000; #4370), and ERK1/2 (1:1000; #4695) were purchased from Cell Signaling Technology; Goat polyclonal antibody against Gadd45b (for IF, 1:500; #sc8776) was purchased from Santa Cruz Biotechnology; Rabbit polyclonal antibody against Microtubule-associated protein 2 (MAP2, 1:100; #17490-1-AP) and mouse monoclonal antibody against glial fibrillary acidic protein (GFAP, 1:500; #60190-1-Ig) were purchased from Proteintech; Mouse monoclonal antibody against GFP (1:200; AG281) was purchased from Beyotime; Mouse monoclonal antibody against Tubulin was purchased from Zhengneng Biotechnology. ProLong® Gold antifade reagent (#1620476) was purchased from Life technologies.

### Primary cortical neurons culture

Primary cerebral cortex neuronal cultures were obtained from Sprague–Dawley rats embryos at embryonic day 17 as described previously [27, 28]. In brief, the cortical tissues were dissected, treated with 0.125 % trypsin solution and 0.05 % DNase I, dissociated by repeated pipetting, and filtered through a Falcon cell strainer (70 μm). Dissociated cortex cells were plated at a density of 1.5 × 10^6^ cells/well on 6-well plates, and cultured in Neurobasal medium supplemented with 2 % B27, 0.25 % GlutaMax, 100U/ml penicillin/streptomycin at 37 °C in a humidified 95 % air and 5 % CO_2_ incubator. Wells were pretreated by incubation with PDL (0.1 mg/ml) at 37 °C for 4 h and then carefully rinsed with phosphate-buffered saline (PBS, pH 7.2) twice. Experiments were performed in vitro Days 7–12 (DIV 7–12), after initial plating of cultures. Neurons were identified by staining with neuronal marker MAP2 and neuroglia maker GFAP antibodies. More than 90 % of primary cultured cortical cells were positive for neuronal marker MAP2, determined by immunohistochemistry and inverted fluorescence microscopy. All experimental procedures were in accordance with the Guide for Care and Use of Laboratory Animals and China Council on Animal Care. Ethical approval was obtained from the Animal Ethics Committee of Chongqing Medical University.

### Oxygen-glucose deprivation (OGD) followed by reperfusion (R)

For an in vitro model of hypoxic/ischemic insult, primary cortical neurons were subjected to moderate oxygen-glucose deprivation and reperfusion, performed following the method as previously described [29]. On DIV 7, while the culture medium was replaced with a glucose-free DMEM, cells were maintained in an anaerobic chamber (0.3 % O_2_; 5 % C0_2_) for 3 h. After 3 h of OGD treatment, the cell culture medium was changed back to normal Neurobasal medium and returned to normoxic conditions for reperfusion (R) 0, 6, 24, 48, 72, and 120 h. In the control condition, cells were treated identically except that they were not exposed to OGD/R.

### Lentiviral vectors construction and lentiviral infection

Silencing Huwe1 lentiviral vector (pGIPZ system) and silencing Gadd45b lentiviral vector (plko1 system) were ordered from Open Biosystem. Third generation pseudotype lentivirus expressing green fluorescent protein (GFP) shRNA Huwe1 (lentiviral-GFP-Huwe1) and expressing shRNA Gadd45b (lentiviral-Gadd45b) were generated as previously described [30, 31]. Probe sequence for shRNA targeted to the coding sequence of Gadd45b and Huwe1 (Gadd45b: Genebank NC_005106.4, TGAAGAGAGCAGAGGCAATAA, Huwe1: Genebank NC_005120.4, TCTAGTAGCCAAATTGGAG) produced with a three-plasmid system by transient transfection of 293FT cells. pCMVdr-8.91 and pMD2G were used as package system. Briefly, for lentivirus production, HEK 293FT cells plated to 70 % confluency were cotransfected with Lipofectamine^®^ 3000 Transfection Reagent. Medium was collected at 48 and 72 h after transfection, and filtered at 0.45 μm pore size and concentrated by ultracentrifugation at 27,000 r.p.m. for 2.5 h. The resulting pellets were re-suspended in 150 μl PBS, pooled and stored at -80 °C. Efficiency of infection as measured by GFP fluorescence was >90 % for the lentiviral-GFP-Huwe1. The measurement of knockdown expression in infected 293FT cells were performed by real-time quantitative PCR using SYBR Green detection as described previously for all lentiviruses.

Knockdown efficiency of lentivirus shRNA-Huwe1 and lentivirus shRNA-Gadd45b was also assessed on primary cortical neurons. Primary cell cultures were infected at the indicated DIV by adding concentrated lentivirus at a multiplicity of infection (m.o.i.) of 0.5 to the growing media. In brief, on DIV 3, three in four of the culture medium was removed from culture wells, and add lentivirus (100 μl shRNA-Huwe1 or 100 μl shRNA-Gadd45b), 500 μl fresh normal culture medium and 5 μg/ml polybrene per well of 6-well plates. The cells were incubated for 24 h at 37 °C, and the removed medium was then returned to the well. Cultures were incubated for a further 4 days before subjected to OGD/R. Transduction efficiency was examined by western blot and quantitative real time PCR for Huwe1 and Gadd45b levels. Lentiviral-scramble-GFP or lentiviral-scramble was used as a control in each experiment to compare to treatment with lentiviral-GFP-Huwe1 or lentiviral-Gadd45b.

### Neuron cell treatment with cycloheximide

Protein stability was measured in the presence of Cycloheximide (CHX). On DIV 3, neuron cells were treated with lentivirus shRNA-Huwe1 or lentivirus GFP-Scramble as descried previously. On DIV 7, neuron cells were subjected to OGD 3 h and reperfusion 24 h, and then treated with CHX (50 μg/ml per well of 6-well plates) and harvested at the indicated times (0, 15, 30, 60 min) followed by western blot for Gadd45b.

### Cell viability study and LDH release assay

Cell viability was evaluated by the CCK-8 assay. Cell death was determined by the LDH into the media, for LDH is an indicator for cell injury. Thus, CCK-8 and LDH activity in the medium after OGD/R was determined according to the protocols of the manufacturer’s manual.

### Hoechst 33342 staining

To perform Hoechst 33342 staining, neurons on 24 mm × 24 mm coverslips (4 × 10^5^ cells/well on 12-wells plate), were fixed in 4 % paraformaldehyde and incubated for 10 min at room temperature with 5 μg/ml Hoechst 33342. The neurons with clearly condensed and segmented chromatin were considered to be apoptotic. For all the groups, 300 cells (including the normal and apoptotic cells) were counted in a blind manner using inverted fluorescent microscopy (Nikon).

### Protein Co-immunoprecipitation

The role of Huwe1 on Gadd45b was detected by Co-Immunoprecipitation (CO-IP) assay. Co-IP assay was conducted according to the protocols of the manufacturer’s manual. Briefly, cells were washed with ice-cold PBS and lysed by ice-cold lysis buffer. Equivalent amounts of neuronal cell protein lysate were mixed with 1 μg the anti-Huwe1 or anti-Gadd45b or anti-IgG antibodies at 4 °C overnight, and then incubated with magnetic beads by rotating mixer at room temperature for 10 min. IgG was used as a negative control. The antibody-bound beads were washed 3 times with PBS/Tween, and placed on a magnet to remove the supernatant by gentle pipetting. The immune-complexes were degenerated with SDS-PAGE sample buffer, and then subjected to Western blot using the indicated antibodies. Band intensities were quantified by densitometry using Image J software.

### Confocal fluorescence microscopy

For colocalization experiments, neuron cells on coverslips were fixed in 4 % paraformaldehyde (PFA) and then incubated with anti-Huwe1 and anti-Gadd45b antibodies at 4 °C overnight. Immunofluorescence labeling was performed following a standard procedure described. After labeling with the primary antibodies, cells were treated with Alexa 594-labeled goat anti-mouse IgG and Alexa 488-labeled goat anti-rabbit IgG secondary antibodies at room temperature for 2 h, washed with PBS, incubated with 4, 6-diamidino-2-phenylindole (DAPI) for nuclear stain, and then mounted with ProLong^®^ Gold antifade reagent. Images were obtained by a confocal laser scanning microscopes (Leica Microsystems). Digital images were recorded and analyzed using FV10-ASW 3.1 Viewer and Image Pro Plus software.

### BDNF DNA methylation analysis

DNA was extracted from neuron cells and analyzed using BSP bisulfite sequencing (BSP). DNA methylation analysis was conducted using standard procedures. The CpG-rich region of the BDNF Exon IXa between 1 and 471 bp (accession number: EF125690.1) includes six CpG sites. PCR primers for amplification of specific targets sequences are listed as follows: 5’-ATGTGTGATTGTGTTTTTGGTG-3’ (sense), 5’-CCTACAAAAAACACAAATCAAT ATC-3’ (anti-sense). For sequence analysis, the BSP products were purified and subjected to direct sequencing in Sangon Biotech (Shanghai, China).

### The activity of Rho assay

The activity of Rho was evaluated by the G-LISA^®^ RhoA Activation Assay Biochem Kit™ (Absorbance Based). The RhoA G-LISA^®^ kit contains a Rho-GTP-binding protein linked to the wells of a 96 well plate. Active, GTP-bound Rho in cell lysates will bind to the wells while inactive GDP-bound Rho is removed during washing steps. The bound active RhoA is detected with a RhoA specific antibody. The degree of RhoA activation is determined by comparing readings from activated cell lysates versus non-activated cell lysates. Inactivation of RhoA is generally achieved in tissue culture by a serum starvation step. Thus, Rho activity in the neuron cells was determined according to the protocols of the manufacturer’s manual.

### Immunofluorescence

For double immunofluorescence, cells on coverslips were fixed in 4 % PFA, treated with 0.1 % Triton X-100 in PBS for 15 min at room temperature, and incubated with 3 % BSA blocking solution for 30 min at 4 °C. Cells were incubated with anti-Gadd45b and anti-BDNF antibodies at 4 °C overnight, washed with PBS, incubated with fluorogenic secondary antibodies for 2 h at room temperature, and then incubated with DAPI. Cells were mounted with ProLong^®^ Gold antifade reagent. Images were obtained by an inverted fluorescence microscope (Nikon).

### Western blot analysis

Extracted total proteins were incubated with cold RIPA lysis buffer and separated by 10 % or 15 % SDS-polyacrylamide gel electrophoresis. The proteins were transferred to 0.22 μm (Bio-Rad) or 0.45 μm (Millipore) PVDF membranes, and reacted with primary antibodies specific to Huwe1, Gadd45b, BDNF, p75, TrkB, p-CREB, CREB, p-ERK1/2, and ERK1/2 at 4 °C overnight. The membranes were washed with TBST, and incubated with secondary antibodies conjugated to horseradish peroxidase. The protein levels were normalized to Tubulin. Bound antibodies on the membrane were detected using an enhanced chemiluminescence detection system (Millipore) according to the manufacturer’s manual. The intensities of bands on blots were analyzed by the Image J software.

### Quantitative real time PCR analysis

SYBR green-based quantitative real time PCR (qPCR) was used to examine the mRNA levels of Huwe1, Gadd45b and BDNF in RNAs prepared from cells. Total RNAs were isolated using Trizol reagent according to the manufacturer’s instructions. qPCR was performed on an ABI 7500 sequence detection system (Life technologies). The GADPH gene was used as an endogenous control. The amount of gene expression was then calculated as the difference cycle threshold (ΔCT) between the CT value of the target gene and the GAPDH. ΔΔCT were difference between ΔCT values of test sample and the control. Relative expression of target genes was calculated as 2 − ΔΔCT. Real time RT-PCR primers as follows:

- Huwe1: forward: 5’-CAAGTAGCCATCAGCAAGA-3’
  - reverse: 5’-GTCCTCCAGTTCATTCTCAA-3’
- Gadd45b: forward: 5’-GTCACCTCCGTCTTCTTG-3’
  - reverse: 5’-GATTCAGTCACACTTCACAG-3’
- BDNF: forward: 5’- CTTAGGCAGAATGAGCAATG-3’
  - reverse: 5’-AATGGACAGAACTATCAGGAG-3’
- GADPH: forward: 5’-GCCAAAAGGGTCATCATCTC-3’
  - reverse: 5’-GTAGAGGCAGGGATGATGTTC-3’

### Statistical analysis of data

Data were expressed as the means ± SD. The comparisons of LDH and CCK-8, and the immunoblot analyses data at different testing time between groups were compared by using Student’s t tests (for comparison between two groups) or one-way ANOVA followed by the Tukey test (for comparison of three or more groups). The differences between two groups were tested with the post hoc least significant difference test. A value of *P* < 0.05 was considered statistically significant. All statistical analyses were conducted using the software Graph Pad Prism Version5.0.

## Additional file

Additional file 1:
**Figure S1.** The expression of Huwe1 and Gadd45b after treatment with shRNA-Huwe1-1 or shRNA-Huwe1-2. The shRNA-Huwe1 was mentioned in our article previously, labeled as shRNA-Huwe1-1. Another probe sequence for shRNA targeted to the coding sequence of Huwe1 was as follows: AATTGCTATGTCTCTGGGACA, labeled as shRNA-Huwe1-2. *a* Neuron cells were treated with lentivirus shRNA-Huwe1-1 or shRNA-Huwe1-2 on DIV 3, and then exposed to OGD 3 h and reperfusion for 24 h on DIV 7. Cells were prepared for western blot and qPCR. Note that western blot assay of Huwe1 and Gadd45b expression at the 24 h after treatment with shRNA-Huwe1-2. *b* Note that qPCR result of Huwe1 and Gadd45b expression at the 24 h after treatment with shRNA-Huwe1-2. *c* Normal neuron cells were treated with lentivirus shRNA-Huwe1-1 or shRNA-Huwe1-2 on DIV 3, and prepared for western blot and qPCR on DIV 7. Note that western blot assay of Huwe1 and Gadd45b expression in normal neuron cells after treatment with shRNA-Huwe1-1 or shRNA-Huwe1-2. *d* Note that qPCR result of Huwe1 and Gadd45b in normal neuron cells after treatment with shRNA-Huwe1-1 or shRNA-Huwe1-2.* Tubulin was used as an internal control for western blot; GADPH was used as an internal control for qPCR. *p<0.05 versus control, #p<0.05 versus LV-GFP-ctrl*. (DOCX 35 kb)

## Abbreviations

BDNF: Brain derived neurotrophic factor
BSP: Bisulfite sequencing PCR
CHX: Cycloheximide
CREB: cAMP response element-binding protein
Gadd45b: Growth arrest and DNA-damage inducible protein 45 beta
OGD/R: Oxygen-glucose deprivation and reperfusion
p75NTR: Nerve growth factor receptor 75
p-CREB: phosphorylated cAMP response element-binding protein
TrkB: Tropomyosin-related kinase B receptors
UPS: Ubiquitin-proteasome system
