# Supplementary material for: Huwe1 interacts with Gadd45b under oxygen-glucose deprivation and reperfusion injury in primary Rat cortical neuronal cells
Source: Mol Brain. 2015 Dec 23;8:88. doi: 10.1186/s13041-015-0178-y (PMC4690333; doi:10.1186/s13041-015-0178-y)
Supplement: Additional file 1: — Figure S1. The expression of Huwe1 and Gadd45b after treatment with shRNA-Huwe1-1 or shRNA-Huwe1-2. The shRNA-Huwe1 was mentioned in our article previously, labeled as shRNA-Huwe1-1. Another probe sequence for shRNA targeted to the coding sequence of Huwe1 was as follows: AATTGCTATGTCTCTGGGACA, labeled as shRNA-Huwe1-2. a Neuron cells were treated with lentivirus shRNA-Huwe1-1 or shRNA-Huwe1-2 on DIV 3, and then exposed to OGD 3 h and reperfusion for 24 h on DIV 7. Cells were prepared for western blot and qPCR. Note that western blot assay of Huwe1 and Gadd45b expression at the 24 h after treatment with shRNA-Huwe1-2. b Note that qPCR result of Huwe1 and Gadd45b expression at the 24 h after treatment with shRNA-Huwe1-2. c Normal neuron cells were treated with lentivirus shRNA-Huwe1-1 or shRNA-Huwe1-2 on DIV 3, and prepared for western blot and qPCR on DIV 7. Note that western blot assay of Huwe1 and Gadd45b expression in normal neuron cells after treatment with shRNA-Huwe1-1 or shRNA-Huwe1-2. d Note that qPCR result of Huwe1 and Gadd45b in normal neuron cells after treatment with shRNA-Huwe1-1 or shRNA-Huwe1-2. Tubulin was used as an internal control for western blot; GADPH was used as an internal control for qPCR. *p<0.05 versus control, #p<0.05 versus LV-GFP-ctrl. (DOCX 35 kb) [file 13041_2015_178_MOESM1_ESM.docx]

In the supplement experiment, another probe sequence for shRNA targeted to the coding sequence of Huwe1 was as follows: AATTGCTATGTCTCTGGGACA, labeled as shRNA-Huwe1-2. So, the shRNA-Huwe1 was mentioned in our article previously, labeled as shRNA-Huwe1-1. Lentiviral vector was produced as previously described. We firstly examined the expression of Huwe1 and Gadd45b at 24 h reperfusion after treatment with shRNA-Huwe1-2. Treatment with shRNA-Huwe1-2 obviously decreased the expression of Huwe1 and increased the expression of Gadd45b at 24 h (Fig s1-a). ShRNA-Huwe1-2 had no effect on the mRNA level of Gadd45b at 24 h (Fig s1-b). We next examined the expression of Huwe1 and Gadd45b in normal cortex neuron cells after treatment with shRNA-Huwe1-1 or shRNA-Huwe1-2. Treatment with shRNA-Huwe1-1 or shRNA-Huwe1-2 decreased the expression of Huwe1 and increased the expression of Gadd45b in normal cortex neuron cells (Fig s1-c). ShRNA-Huwe1-1 or shRNA-Huwe1-2 had no effect on the mRNA level of Gadd45b in normal neuron cells (Fig s1-d).

**Figure legends**

**Fig s1** **The expression of Huwe1 and Gadd45b after treatment with shRNA-Huwe1-2.** Neuron cells were treated with lentivirus shRNA-Huwe1-1 or shRNA-Huwe1-2 on DIV 3, and then exposed to OGD 3 h and reperfusion for 24 h on DIV 7. Cells were prepared for western blot and qPCR. *a* Western blot assay of Huwe1 and Gadd45b expression at the 24 h after treatment with shRNA-Huwe1-2. *b* QPCR result of Huwe1 and Gadd45b expression at the 24 h after treatment with shRNA-Huwe1-2. *c* Western blot assay of Huwe1 and Gadd45b expression in normal neuron cells after treatment with shRNA-Huwe1-1 or shRNA-Huwe1-2. *d* qPCR result of Huwe1 and Gadd45b in normal neuron cells after treatment with shRNA-Huwe1-1 or shRNA-Huwe1-2. *Tubulin was used as an internal control for western blot; GADPH was used as an internal control for qPCR.*p<0.05 versus control,* ***^#^****p<0.05 versus LV-GFP-ctrl.*
